# Identification of a 4‐mRNA metastasis‐related prognostic signature for patients with breast cancer

**DOI:** 10.1111/jcmm.14049

**Published:** 2018-11-28

**Authors:** Xinhua Xie, Jianwei Wang, Dingbo Shi, Yutian Zou, Zhenchong Xiong, Xing Li, Jianhua Zhou, Hailin Tang, Xiaoming Xie

**Affiliations:** ^1^ Department of Breast Oncology State Key Laboratory of Oncology in South China Collaborative Innovation Center for Cancer Medicine Sun Yat‐sen University Cancer Center Guangzhou China; ^2^ Department of Ultrasond State Key Laboratory of Oncology in South China Collaborative Innovation Center for Cancer Medicine Sun Yat‐sen University Cancer Center Guangzhou China; ^3^ Department of Experimental Research State Key Laboratory of Oncology in South China Collaborative Innovation Center for Cancer Medicine Sun Yat‐sen University Cancer Center Guangzhou China

**Keywords:** breast cancer, mRNAs signature, prognosis, sisease‐free survival

## Abstract

Metastasis‐related mRNAs have showed great promise as prognostic biomarkers in various types of cancers. Therefore, we attempted to develop a metastasis‐associated gene signature to enhance prognostic prediction of breast cancer (BC) based on gene expression profiling. We firstly screened and identified 56 differentially expressed mRNAs by analysing BC tumour tissues with and without metastasis in the discovery cohort (GSE102484, n = 683). We then found 26 of these differentially expressed genes were associated with metastasis‐free survival (MFS) in the training set (GSE20685, n = 319). A metastasis‐associated gene signature built using a LASSO Cox regression model, which consisted of four mRNAs, can classify patients into high‐ and low‐risk groups in the training cohort. Patients with high‐risk scores in the training cohort had shorter MFS (hazard ratio [HR] 3.89, 95% CI 2.53‐5.98; *P* < 0.001), disease‐free survival (DFS) (HR 4.69, 2.93‐7.50; *P* < 0.001) and overall survival (HR 4.06, 2.56‐6.45; *P* < 0.001) than patients with low‐risk scores. The prognostic accuracy of mRNAs signature was validated in the two independent validation cohorts (GSE21653, n = 248; GSE31448, n = 246). We then developed a nomogram based on the mRNAs signature and clinical‐related risk factors (T stage and N stage) that predicted an individual's risk of disease, which can be assessed by calibration curves. Our study demonstrated that this 4‐mRNA signature might be a reliable and useful prognostic tool for DFS evaluation and will facilitate tailored therapy for BC patients at different risk of disease.

## 1 INTRODUCTION

Breast cancer (BC) has become a main health burden owing to the high rates of morbidity and cancer‐related mortality among women.^1^, ^2^, ^3^ Currently, comprehensive treatment strategy for BC, such as chemotherapy, radiotherapy and target therapy, mainly depends on the tumour stage and molecular subtypes.^4^, ^5^, ^6^, ^7^ Up to now, the American Joint Committee on Cancer (AJCC) staging system has always been broadly adopted for cancer management, including BC.^4^, ^5^, ^8^, ^9^ However, the current TNM Classification method cannot perfectly provide accurate information to predict patients’ prognosis.^10^, ^11^ Then some patients with BC received unnecessary or excessive medication, while others may be faced with recurrence or metastasis due to lack of appropriate treatment.^12^, ^13^ These limitations have prompted a search for new biomarkers for discrimination of cancer patients to improve precision cancer treatment.

In recent years, detailed information regarding prognosis evaluation for cancer patients can be effectively provided by genome‐wide expression profiling detection.^14^, ^15^, ^16^, ^17^ Numerous studies have evaluated the prognostic roles of array‐based gene expression signatures acquired from tumours.^14^, ^18^, ^19^ Several gene signatures have also been established to distinguish the prognosis of patients beyond the BC clinicopathologic features; however, most of them are not used clinically.^17^, ^19^, ^20^ Thus, identifying a novel and practical gene signature to predict patients’ prognosis is urgently needed and of great clinical significance.

Several studies have identified that a number of mRNAs are differentially expressed in nasopharyngeal carcinoma,^21^ lung cancer^22^ and colorectal cancer^23^ and so on,^24^ which are associated with survival prognosis. As mRNAs related to survival are usually associated with the development and metastasis of some cancers, it is vital to develop a robust BC prognosis‐related mRNAs signature. Therefore, to identify mRNAs that might serve as potentially accurate markers for predicting clinical outcome, we used the Gene Expression Omnibus (GEO) database to characterize the mRNAs profiling on large cohorts of BC patients, and finally identified a 4‐mRNAs signature which can be validated.

## 2 METHODS

### 2.1 Patient cohorts

To overcome the bias in microarray platforms, this study only included patients from BC‐related gene expression datasets measured by the same platform (GPL570‐55999, the Affymetrix HU133 Plus 2.0 microarray), including GSE102484, GSE20685, GSE21653 and GSE31448. All these gene expression and corresponding clinical data were obtained from the GEO database (https://www.ncbi.nlm.nih.gov/geo), so the approval of ethics committee was not needed. To evaluate the correlations of mRNAs expression with survival status for BC patients, we screened those datasets that included more than 200 patients with disease‐free survival (DFS) for model development and validation in this study. In total, 1496 samples (683 from GSE102484, 319 from GSE20685, 248 from GSE21653 and 246 from GSE31448) were obtained (Table 1). The GSE102484 cohort was used as the discovery cohort, and then the GSE20685 cohort was applied for the training cohort. In addition, we randomly consider GSE21653 and GSE31448 as independent external validation cohorts. A summary of these cohorts and procedures for data processing are provided in Supplementary Methods.

**Table 1:** Summary of BC‐related mRNAs expression datasets and corresponding clinical characteristics

Characteristic	GSE102484 (n = 683)	GSE20685 (n = 319)	GSE21653 (n = 248)	GSE31448 (n = 246)
Age (years)				
≤40	119 (17.42)	80 (25.08)	40 (16.13)	40 (16.26)
>40	564 (82.58)	239 (24.92)	208 (83.87)	206 (83.74)
T stage				
T1‐2	653 (95.60)	289 (90.60)	178 (71.77)	178 (72.36)
T3‐4	30 (4.40)	30 (9.40)	63 (25.40)	62 (25.20)
N stage				
N0	300 (43.92)	137 (42.95)	116 (46.77)	115 (46.75)
N1‐3	383 (56.08)	182 (57.05)	130 (52.42)	129 (52.44)
Survival status				
Metastasis‐free	582 (85.21)	244 (76.49)		
Metastasis	101 (14.79)	75 (23.51)		
Disease‐free		233 (73.04)	169 (68.15)	167 (67.89)
Disease		76 (23.82)	79 (31.85)	79 (32.11)

### 2.2 Study design

#### 2.2.1 Discovery cohort

**Figure 1:**
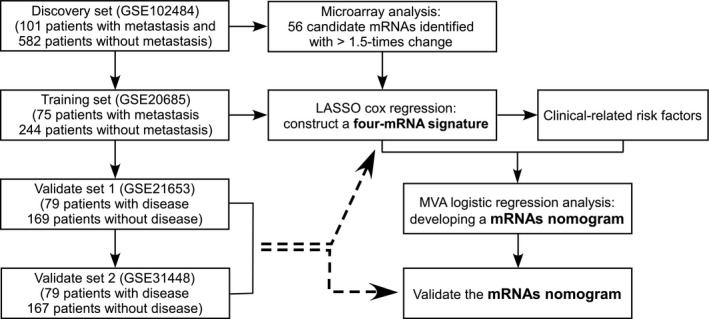
Study design for the identification of BC survival‐related 4‐mRNA signature

Differential mRNAs expression analysis by array was used to identify mRNAs differentially expressed in primary tumour tissues of patients with BC that have developed distance metastases or not. To screen mRNAs expression profiles, 683 tumour samples were obtained from BC patients, which included 101 patients with distance metastasis and 582 patients without distance metastasis (Figure 1, Table 1).

#### 2.2.2 Model development and validation cohorts

A total of 319 BC specimens from GSE20685 were obtained for the training cohort to develop mRNAs signature model. Fifty‐six candidates differentially expressed mRNAs screened from discovery set were used to construct mRNAs signature. Firstly, we analysed the prognostic role of the candidate mRNAs and found 26 mRNAs were associated with distant metastasis‐free survival (MFS) in the training cohort; Then, collinearity analysis was taken for the 26 mRNAs; Thirdly, we used LASSO Cox regression model to select prognostic mRNAs to predict the MFS of the patients with 1000 bootstrap replicates. The ultima risk score = C1X1 + C2X2 + C3X3 + ……CnXn (c is the regression coefficients obtained from the Cox analysis and X is the expression value of each mRNAs). Then, the patients were classified into high and low risk based on the optimal cut‐off value of the predictor score, which was selected based on X‐tile software (version 3.6.1, Yale University, CT). For the validation process, 494 patients from GSE21653 and GSE31448 were considered to evaluate the expression values of mRNAs signature respectively. Moreover, prognostic nomogram based on mRNAs signature and clinical‐related variables was plotted. In addition, patients’ inclusion and exclusion criteria for training cohort and validation cohorts are showed in the Supplementary Methods.

### 2.3 Statistical analysis

All the statistical analysis was performed with R software (version 3.2.3; http://www.Rproject.org) and the SPSS software (version 22; SPSS Inc., Chicago, IL, USA). The conventional two‐sided tests, and a significance level of 0.05 were used in all analyses. We used the LASSO cCox regression model to select the most useful prognostic molecular markers of all the metastasis‐associated mRNAs identified in the training set, and constructed a mRNAs‐based classifier for predicting the survival status of patients with BC in the training cohort. Survival times were compared between the two groups by using the Kaplan‐Meier analysis at a *P*‐value <0.05. Nomogram was plotted using the rms package in R, and included pT stage and pN stage in the nomogram as they are usually included in most prognostic models of BC. We used the coefficients of the multivariable Cox regression model to depict nomogram. The performance of the prediction model developed using the training cohort was validated by assessing the calibration curves.

## 3 RESULTS

### 3.1 The expression of cancer metastasis‐associated mRNAs in datasets through array re‐annotation

Because a number of large‐scale gene expression datasets are available in the GEO database, an integrative analysis of re‐annotated mRNA expression datasets would provide good statistical power to capture the expression changes of mRNAs in disease condition. To avoid the inconsistency of the mRNAs expression levels on different platforms, we only collected datasets measured on the Affymetrix HU133 Plus 2.0 microarray platform to identify potential mRNAs prognostic biomarkers. After a thorough search of the GEO database, we identified three gene expression datasets with DFS time (GSE20685, GSE21653 and GSE31448). Together, these data sets include a total of 891 BC patient samples (Table 1). Next, the corresponding mRNA expression datasets were constructed using array re‐annotation analysis as described in the Methods section. Relevant clinical information including age, tumour stage (T stage), axillary lymph node stage (N stage) and DFS for the four mRNAs expression datasets are summarized in Table 1.

### 3.2 Development of survival‐associated mRNAs signature for patients with BC

**Figure 2:**
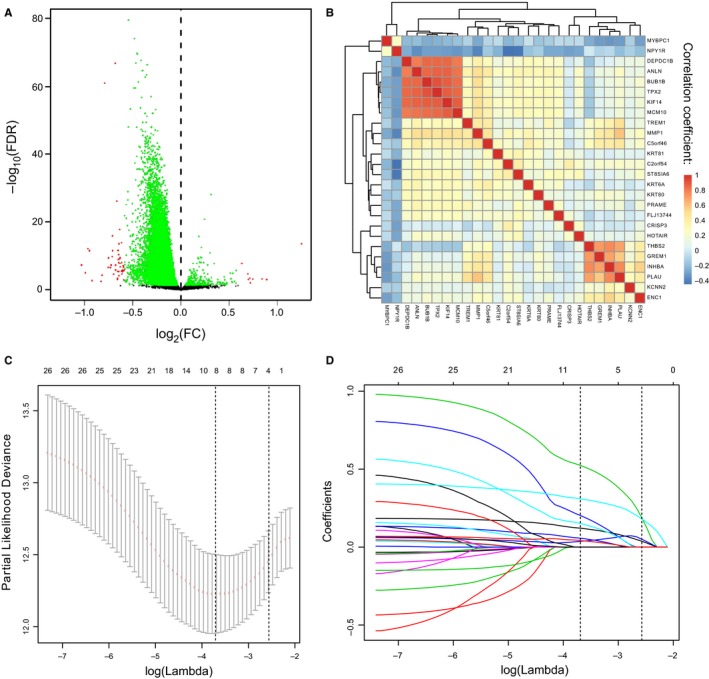
Construction of a 4‐mRNA signature. (A) Fifty‐six mRNAs selected by univariate analysis. Volcano plot illustrating a comparison of mRNAs expression in BC with metastasis‐free and metastasis. (B) Hierarchical clustering shows the collinearity of 26 candidate mRNAs. (C) LASSO algorithms was used to identify and evaluate the 4‐mRNA signature in the training cohort. Four mRNAs were selected and used to develop a 4‐mRNA signature to predict patients prognosis. (D) LASSO coefficient profiles of the 26 metastasis and prognostic‐related mRNAs based on the training data (GSE20685)

**Figure 3:**
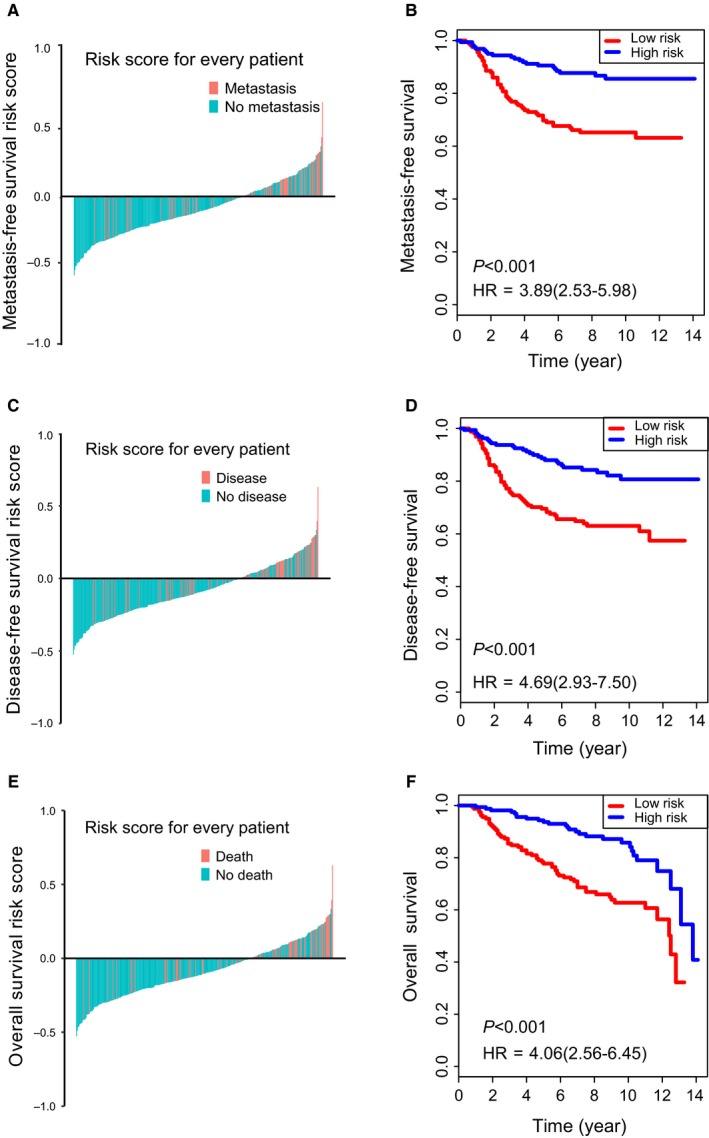
Analysis of the 4‐mRNA signature in the training cohort. The distribution of patients’ risk score and metastasis (A), disease (C) or death status (E); Kaplan‐Meier survival curves of MFS (B), DFS (D) and OS (F) between high‐risk and low‐risk patients in GSE20685

Samples in discovery cohort were divided into metastasis group and non‐metastasis group. Fifty‐six mRNAs were found to be differentially expressed between the two groups (*P* < 0.05, fold change ≥1.25; Figure 2A). To identify the survival‐associated mRNAs, we conducted Cox regression analysis and found 26 of these differentially expressed genes were associated with MFS in the training cohort. We further proceeded collinear analysis and found collinearity among some mRNAs, which may prejudice the accuracy of traditional Cox regression analysis (Figure 2B). Therefore, we used LASSO Cox regression method and finally identified four mRNAs from the 26 differentially expressed mRNAs with the highest frequency of being selected by this method among 200 bootstrap replicates, which were as follow: KCCN2, cysteine‐rich secretory protein 3 (CRISP3), GREM1 and KRT80 (Figure 2C). A coefficient profile plot produced for the 26 survival‐associated mRNAs are shown in Figure 2D. Using LASSO Cox regression results, we derived a 4‐mRNA signature to calculate the risk score for every BC patient based on the expression levels of these 4 mRNAs weighted by their regression coefficients: risk score = 0.1213333 × ×*I*_KCCN2_+02576976 × *I*_CRISP3_+0.03454120 × *I*_GREM1_+0.1575855 × *I*_KRT80_. In this formula, *I*_mRNAx_ indicates the log2‐scaled expression value of mRNA_x_. Patients in the training set with a risk score <2.06 were assigned to the low‐risk group, while those with a score ≥2.06 were assigned to the high‐risk group using the cut‐off point by X‐tile (Supplementary Figures). The distribution of risk scores and survival status is shown in Figure 3A,C,E, which suggested that patients with lower risk scores generally had better survival than those with higher risk scores. The MFS rates for patients with low‐risk scores were 90.3% at 5 years compared with 61.0% in patients with high‐risk scores, respectively (hazard ratio [HR]: 3.89, 95% confidence interval [CI]: 2.53‐5.98, *P* < 0.001, Figure 3B). We then did the same analyses for DFS and overall survival (OS), 5‐year DFS and OS was 59.3% and 68.7% for the high‐risk group and 87.6% and 93.6% for the low‐risk group (HR: 4.69, 95% CI: 2.93‐7.50, *P* < 0.001, Figure 3D; HR: 4.06, 95% CI: 2.56‐6.45, *P* < 0.001, Figure 3F).

### 3.3 Validation of a 4‐mRNA signature to predict DFS of patients with BC

**Figure 4:**
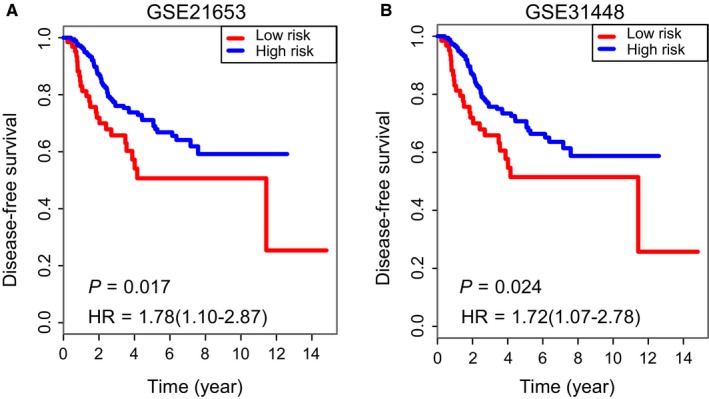
Validation of the 4‐mRNA signature in two independent validation cohorts. Kaplan‐Meier survival curves of DFS between high‐risk and low‐risk patients in (A) GSE21653 and (B) GSE31448

To confirm that the 4‐mRNA‐based classifier had similar prognostic value in different populations, we evaluated the samples in GSE21653 and GSE31448, respectively. Using the established cut‐off point, 185 (74.8%) patients were classified as low risk, and 63 (25.2%) as high risk in GSE21653. The corresponding 5‐year DFS was 56.3% for the high‐risk group and 74.0% for the low‐risk group in GSE21653 (HR: 1.78, 95% CI: 1.10‐2.87; *P* = 0.017; Figure 4A). Similarly, in validation set GSE31448, the 5‐year DFS was 57.7% for the high‐risk group and 73.4% for the low‐risk group (HR: 1.72, 95% CI: 1.07‐2.78; *P* = 0.024; Figure 4B).

### 3.4 Nomogram combined mRNAs signature and clinical‐related variables predicts patients’ DFS

**Figure 5:**
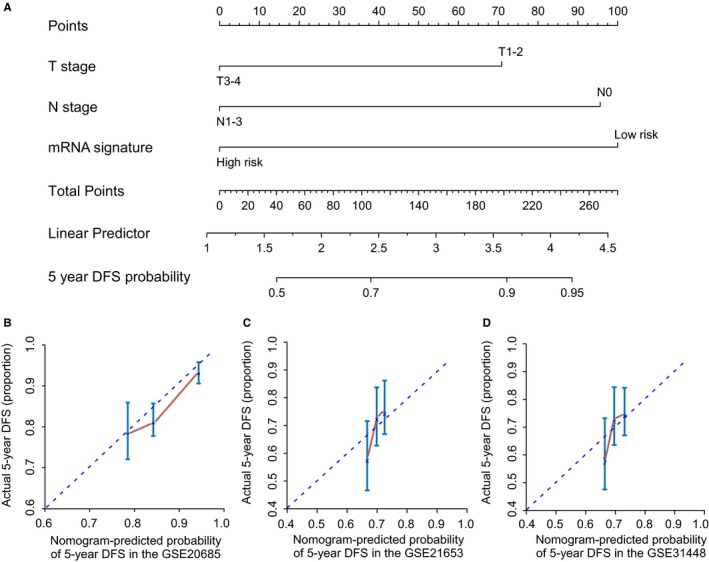
mRNA nomogram to predict the risk of disease in patients with BC. (A) mRNA nomogram to predict disease‐free survival. Calibration curves of the nomogram to predict disease‐free survival at 10 years in (B) GSE20685, (C) GSE21653 and (D) GSE31448. The actual disease‐free survival is plotted on the *y*‐axis; nomogram predicted probability is plotted on the *x*‐axis. DFS: disease‐free survival

In the training cohort, univariate and multivariate analysis all revealed that T stage, N stage and mRNAs signature were significantly associated with DFS (Table 2). Then based on the above analysis results, we developed a mRNAs nomogram that combined the clinical‐related factors (T stage and N stage) and mRNAs signature (Figure 5A; Table 2). The calibration plots for the 5‐year DFS were predicted well in the training cohort, the GSE21653 validation cohorts and the GSE31448 validation cohort (Figure 5B‐E).

**Table 2:** Univariate and multivariate Cox regression analysis in the training set

Variables	Univariate Cox regression		Multivariate Cox regression	
	HR (95% CI)	*P*	HR (95% CI)	*P*
Age (>40 vs ≤40)	0.81 (0.51‐1.30)	0.383		
T stage (T3‐4 vs T1‐2)	3.28 (1.93‐5.60)	<0.001	1.446 (1.036‐2.019)	0.0027
N stage (N1‐3 vs N0)	3.89 (2.26‐6.70)	<0.001	2.849 (1.626‐4.991)	<0.001
mRNAs signature (high risk vs low risk)	3.89 (2.53‐5.98)	<0.001	3.236 (2.095‐4.997)	<0.001

CI: confidence interval; HR: hazard ratio.

## 4 DISCUSSION

An array‐based database to identify survival‐associated mRNAs for prognostic prediction is significant and urgently needed to guide tailored therapy for patients with BC.^20^, ^21^, ^23^ In our study, we used GEO array data to screen differential mRNAs in a discovery set and selected 56 significant mRNAs according to the likelihood of patients with distant metastasis. Then, in the training cohort, based on LASSO method, we identified four candidate mRNAs and developed a 4‐mRNA signature. Moreover, we validated the mRNAs signature in the two external validation cohorts. With this mRNAs signature, patients’ survival may be predicted before system treatment. Moreover, we established a mRNAs nomogram including mRNAs signature and clinical‐related risk factors (T stage and N stage) to predict DFS. The performance of our nomogram was also evaluated in two validation cohorts. Thus, our nomogram may help guide prognosis prediction and make individualized therapeutic decision for patients with BC.

Our study results show that the evaluation of four mRNAs expression could be a vital tool for the management of BC patients, which can guide the stratification of patients that may recur or metastasis, aiding in decision making for tailored therapy, and then ultimately contributing to an improvement in survival rates.

We determined a set of four mRNAs consisting of KCNN2, KRT80, GREM1 and CRISP3 that predicts DFS in three independent patient sets. KCNN2 was found to be involved in the bile secretion, and the abnormal expression of KCNN2 may be closely related to the pathogenesis of cholangiocarcinoma.^25^ In addition, KCNN2 was also found under‐expressed in Ewing's sarcoma family of tumours relative to alveolar rhabdomyosarcomas, and may be also involved in prostate carcinomas^26^; Keratin 80, also known as KRT80,^27^ whose gene is located at the centromeric end of the type II keratin gene domain, are filament proteins that constitutes one of the main structural fibres of epithelial cells. Previous reports show that GREM1^28^ has a proangiogenic function by directly binding to vascular endothelial growth factor receptor 2^29^ and may increase angiogenesis, which is associated with poorer prognosis.^30^ The expression of CRISP3 was associated clinical outcome in prostate cancer.^31^

However, several limitations are still existed in this study as well. First of all, our study is entirely retrospective and inherent biases may influence results. In the second place, clinical‐related factors, such as molecular classification and treatment related information, has not been analysed due to lack of relevant information in the training and validation sets. In addition, the sample size is too small and array data were all obtained from single platform, which may disturb the application of our constructed model. Last but not the least, although mRNAs signature and nomogram showed good predictive accuracy in the training cohort, their performance in the two external validation cohorts is still low and remains to be improved. Therefore, more markers should be mined and incorporated into our prediction model in future.

## 5 CONCLUSION

Taken together, we filtered specific mRNAs differentially expressed between patients with or without distant metastasis and successfully constructed a prognostic associated mRNAs signature which may aid our prognosis prediction and tailored therapy for BC. Importantly, we developed a 4‐mRNA nomogram that incorporated both mRNA signature and clinical‐related risk factors to predict patients’ prognosis. We confirmed this signature could service as potential specificity biomarkers in the prognosis prediction for BC patients.

## CONFLICT OF INTEREST

We gratefully recognize the patients who participated in this study. We declare no conflicts of interest.

## AUTHOR CONTRIBUTION

Conceptualization, Xinhua Xie, Dingbo Shi, Hailin Tang and Xiaoming Xie; Data curation, Yutian Zou, Zhenchong Xiong and Jianhua Zhou; Formal analysis, Xinhua Xie, Jianwei Wang and Dingbo Shi; Funding acquisition, Hailin Tang and Xiaoming Xie; Methodology, Yutian Zou, Zhenchong Xiong and Jianhua Zhou; Project administration, Hailin Tang; Software, Xinhua Xie, Jianwei Wang, Dingbo Shi and Xing Li; Supervision, Xinhua Xie, Hailin Tang and Xiaoming Xie; Validation, Hailin Tang; Visualization, Hailin Tang; Writing‐original draft, Xing Li; Writing‐review & editing, Xinhua Xie, Jianwei Wang, Hailin Tang and Xiaoming Xie.
