# Supplementary material for: Identification of a 4‐mRNA metastasis‐related prognostic signature for patients with breast cancer
Source: J Cell Mol Med. 2018 Nov 28;23(2):1439–47. doi: 10.1111/jcmm.14049 (PMC6349190; doi:10.1111/jcmm.14049)

**Supplementary Data**

**I. Supplementary Methods**

**1. Data processing procedure**

All the microarray raw data (CEL files) of four BC sets were obtained from the GEO database. Raw data were normalised using the Affymetrix package in R with Robust Multi-array Analysis (RMA). Empirical Bayes (eBayes) in the limma package was used to compute which probes were significantly differentially expressed between metastatic and non-metastatic samples. Expression values of one mRNA gene detected by at least four probes were retained. The mean expression value of multiple probes mapping to the same mRNA was used to represent the expression level of the mRNA. For each set, the expression data was log 2 transformed and normalized by the quantile-normalization approach. The edgeR package in R Bioconductor was used to identify differentially expressed mRNA. In this study, significant differences in gene expression were defined by two criteria: fold change≥1.25 and adjusted P value < 0.05.

**2. Inclusion and exclusion criteria**

**Inclusion criteria:** (a) Tumor samples were detected by GPL570-55999 platform (the Affymetrix HU133 Plus 2.0 microarray). (b) Diagnosis of breast cancer. (c) The number of samples was more than 200. (d) Study period was between Jan.2010 and Dec.2017.

**Exclusion criteria included:** (a) preoperative chemotherapy; (b) clinical variation (T stage and N stage) and follow-up information were unavailable for training and validation cohorts.

**II. Supplementary Figures**

**Figure S1: Identification of the optimum cutoff value for the mRNAs signature.**

The optimum cutoff value of the mRNA signature risk scores was determined using the X-tile program in the GSE20685 training cohort. The strength of the association at each division was represented with the colours shown in the plot. Red indicates an inverse association, while green shows a direct association between the risk score and disease-free survival.


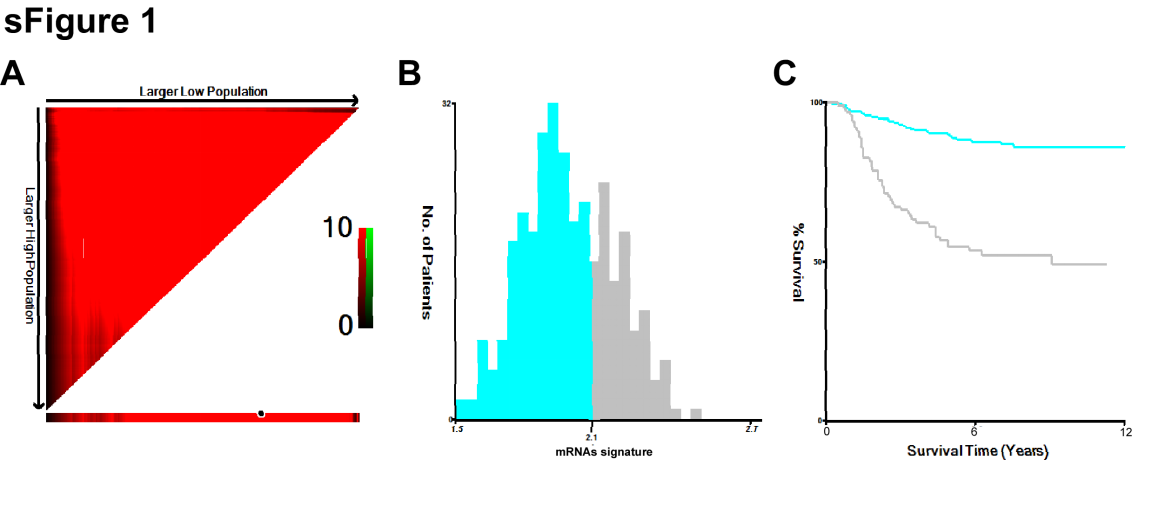

Supplement: Supplementary file 1 [file JCMM-23-1439-s001.doc]
